# Evaluation of the efficacy and safety of nirmatrelvir/ritonavir co-administration inpatients with rheumatic disease infected with SARS-CoV-2: a real-world study

**DOI:** 10.3389/fphar.2023.1288402

**Published:** 2023-12-06

**Authors:** Xue Zhong, Chao Wang, Lin Huang, Yue Zhao, Tianyi Li, Jing He, Xiaohong Zhang

**Affiliations:** ^1^ Department of Pharmacy, Peking University People’s Hospital, Beijing, China; ^2^ Department of Pharmacy, Tianjin First Central Hospital, Tianjin, China; ^3^ School of Pharmaceutical Sciences, Peking University, Beijing, China; ^4^ College of Pharmaceutical Engineering of Traditional Chinese Medicine, Tianjin University of Traditional Chinese Medicine, Tianjin, China; ^5^ Department of Rheumatology and Immunology, Peking University People’s Hospital, Beijing, China

**Keywords:** nirmatrelvir-ritonavir, rheumatic disease, coronavirus disease 2019, severe acute respiratory syndrome coronavirus 2, real-world study

## Abstract

**Background:** The breakthrough development of novel severe acute respiratory syndrome coronavirus-2 (SARS-CoV-2) vaccines and oral antivirals have played a critical role in curtailing the spread of the pandemic and dramatically reducing the morbidity and mortality rates among those infected. Among these oral antivirals, nirmatrelvir/ritonavir (NR) has been repurposed successfully for use against coronavirus disease-2019 (COVID-19) and is now readily available on the market with promising therapeutic effects. The availability of convenient and effective NR treatments for COVID-19 greatly mitigates the severity of the epidemic and contributes to an early end to the pandemic. Furthermore, certain patient subgroups, specifically those with rheumatic disease (RD) who are currently undergoing intensive immunodeficiency and/or immunosuppressive treatments, continue to be vulnerable and at a higher risk of experiencing severe consequences from COVID-19. Additionally, it has also been observed that NR exhibited prevalent drug-drug interactions of clinical significance, and more instances of COVID-19 rebound were being recognized with increasing frequency.

**Methods:** A retrospective cohort study was conducted on a real-world RD population who were infected with SARS-CoV-2 and treated with NR. The time of symptom resolution, length of hospitalization, and response rate were assessed. Results were compared among the standard regimen and non-standard regimen groups, early NR regimen and late NR regimen groups, and the NR indication regimen and NR non-indication regimen groups. During the course, all grades of adverse drug reactions (ADRs) directly associated with NR administration and associated with drug-drug interactions (DDIs) were also monitored.

**Results:** A total of 32 patients with RD, who were infected with SARS-CoV-2 and received NR, were retrospectively identified and divided into different groups. We found that the standard regimen group and the early NR regimen group had a shorter median time of symptom resolution compared to the control group [9.0 (interquartile range [IQR], 8.3-11.3) vs. 21.5 (IQR16.0-24.0) days, *p* < 0.001 and 9.0 (IQR 8.3-11.3) vs. 23.0 (IQR 18.0-24.0) days, *p* = 0.0]. We further found that even if the NR administration time exceeds 5 days, patients with RD who receive the NR indication regimen can still derive certain benefits from it. The proportion of patients who showed symptom improvement was higher in the NR indication regimen compared to the NR non-indication regimen group (n = 13/17 vs. 3/6, 76.5% vs. 50.0%) at the end of follow-up, and there was a statistical difference *(p* = 0.0) in the response rate of patients between the two groups. We also analyzed the effect of comorbidities on patient response rates and found that the percentage of patients who showed symptom improvement was higher in the group with <4 comorbidities compared to the group with ≥ 4 comorbidities (n = 7/7 vs. 16/25, 100.0% vs. 64.0%) at the end of follow-up. During the course, all grades of ADRs and grade ≥3ADRs directly associated with NR administration were not observed in any of the 32 cases. Despite discontinuing warfarin prior to NR application (using NR immediately on the first day of warfarin withdrawal), one patient still experienced an increased international normalized ratio [INR, 5.32(0.90-1.20)] and coagulation disorders (weak positive fecal occult blood test) on the second day after using NR. The INR levels decreased to nearly normal values, and coagulation disorders returned to normal after 2 days of discontinuing NR (the seventh day after the initial administration of NR).

**Conclusion:** We showed NR therapy to be associated with a favorable outcome and an acceptable safety profile in an immunosuppressed population with RD during the Omicron surge. Early use of NR (within 5 days of symptom onset) could improve the prognosis of patients. NR administration for symptoms and confirmed SARS-CoV-2 infection after >5 days may also mitigate progression to severe disease and is a viable strategy. Our results highlight the importance of early utilization and/or NR indication, which may yield clinical advantages for patients with RD infected with SARS-CoV-2.

## 1 Introduction

Since the onset of the COVID-19 pandemic, numerous therapeutic solutions have emerged that have fundamentally transformed the medical landscape of COVID-19. The global community has made concerted efforts to neutralize the replicative capabilities of its causative agent: SARS-CoV-2. Several new and repurposed natural and/or synthetic compounds are undergoing extensive investigations (preclinical studies, clinical trials, and pharmacological evaluations) worldwide as potential efficacious anti-COVID-19 drugs.

Inhibitors targeting key enzymes (e.g., RNA-dependent RNA polymerase, papain-like protease, and main protease) involved in various lifecycle stages of SARS-CoV-2 have been reported. These include CoViTris2020/ChloViD 2020, Taroxaz-26, Taroxaz-104, teriflunomide, azvudine, 2′,3′-dideoxyinosine, forodesine, riboprine, cordycepin, ensitrelvir (S-217622), SLL0197800, CoViTris 2022, and ChloViD 2022 (Rabie, 2021a; Rabie, 2021b; Rabie, 2021c; Zhang et al., 2021; Rabie, 2022; Rabie and Abdalla, 2022; Eltayb et al., 2023; Rabie and Abdalla, 2023; Rabie and Eltayb, 2023; Rabie et al., 2023).

Moreover, several pharmaceutical agents, including chloroquine, hydroxychloroquine, darunavir, arbidolfavir, remdesivir, ribavirin, ritonavir, interferons, dexamethasone, and tocilizumab, have been repurposed for COVID-19 treatment in clinical settings with varying degrees of success. The clinical benefits of monoclonal antibodies targeting the spike protein of SARS-CoV-2 have also been demonstrated in COVID-19 treatment.

The main challenge of antiviral therapies is that they require implementation as soon as possible after SARS-CoV-2 infection to act directly on viral replication—delayed administration of antiviral agents can result in a lack of efficacy (Rahmah et al., 2022). Development of novel SARS-CoV-2 vaccines and oral small-molecule antiviral drugs has played a vital part in curtailing the spread of the pandemic, and reduced the morbidity and mortality rates among those infected. COVID-19 vaccination aims to decrease the prevalence of hospitalization, admission to intensive care units, and death. The drugs stated above simplify infection management and reduce the prevalence of hospitalization in patients with COVID-19 at risk of disease progression (Rahmah et al., 2022; Focosi et al., 2023).

Among these oral small-molecule antiviral drugs, nirmatrelvir/ritonavir (NR) has been repurposed for use against COVID-19. NR is readily available on the market and has promising therapeutic effects. The availability of convenient and efficacious NR treatments for COVID-19 could mitigate the severity of the COVID-19 epidemic and contribute to its early end.

NR received conditional approval from the China National Medical Products Administration on 11 February 2022 thanks to its favorable efficacy and safety profile. However, the application of NR adheres strictly to the principles outlined in the usage instructions and guidelines (U S Food and Drug Administration, 2023; National Health Commission of the People’s Republic of China, 2023; Devresse et al., 2022). Adults who are diagnosed with mild-to-moderate COVID-19, and with a high risk of disease progression, are prescribed NR within 5 days of symptom onset. Studies have consistently confirmed the efficacy of NR in reducing the severity and mortality of COVID-19 when following the aforementioned administration instructions.

In the EPIC-HR trial (Hammond et al., 2022), which evaluated protease inhibition in COVID-19 for high-risk patients, NR administration resulted in significantly fewer COVID-19-related hospitalizations or deaths by day 28 when compared with the placebo group. The relative risk reduction was 89.1% and 88.9% at the interim and final analysis time points, respectively. However, the randomized controlled trials endorsing the use of NR in phases II/III were conducted before the emergence of Omicron variants, which are currently almost 100% prevalent. Those trials involved unvaccinated patients with COVID-19 and excluded individuals with rheumatic disease (RD) (Dal-Ré et al., 2022; Gerolymatou et al., 2023). Furthermore, certain patient subgroups (specifically those with RD undergoing intensive immunodeficiency and/or immunosuppressive treatments) continue to be vulnerable and are at a higher risk of experiencing severe consequences from COVID-19. In terms of treatment and prognosis, patients with COVID-19 with RD may benefit from certain disease-modifying antirheumatic drugs (DMARDs), but solid evidence to support this postulation is lacking (Gianfrancesco et al., 2020; Alzahrani et al., 2021; Santos et al., 2021; Oztas et al., 2022; Pehlivan and Aydin, 2022; Rabie et al., 2022). Whether NR is a safe and efficacious treatment method in patients suffering from RD warrants investigation. In addition, NR has exhibited drug-drug interactions (DDIs) of clinical importance, and instances of “COVID-19 rebound” are being recognized with increasing frequency (Marzolini et al., 2022). Hence, post-marketing assessments and updated real-world data regarding the efficacy and safety of NR have become increasingly important. Herein, we detail our experience of the efficacy and safety of NR in patients suffering from RD.

## 2 Materials and methods

### 2.1 Ethical approval of the study protocol

The study protocol was approved (2021PHB047-001) by the Ethics Committee of Peking University People’s Hospital (Beijing, China) and complied with the Declaration of Helsinki 1964 and its later amendments.

### 2.2 The inclusion/exclusion criteria of the population

This was a real-world study conducted at the Department of Rheumatology and Immunology within Peking University People’s Hospital. We retrospectively selected patients aged ≥18 years with pre-existing RD who were infected with SARS-CoV-2 and received NR treatment between 8 December 2022 and 13 January 2023. We selected this start date with the aim of reducing selection bias by ensuring most cases were caused by the Omicron variant. The diagnosis of COVID-19 was based on a positive test for the nucleic acids of SARS-CoV-2 as well as clinical manifestations, laboratory tests, and imaging (Rabie, 2021b). All patients were administered antiviral therapy using NR after hospital admission. They were closely monitored from hospital admission to hospital discharge or death.

Patients receiving any other form of antiviral therapy, who previously had NR treatment, or who received NR outside the hospital setting were excluded.

### 2.3 Data collection

We identified patients suffering from RD with COVID-19 using an electronic medical record (EMR) system. Certain information was extracted retrospectively from the EMR of each patient: demographic characteristics (age, ethnicity/race, sex); date of hospital admission; RD-related diagnoses; comorbidities; primary immunosuppression or immunomodulation regimens for specific RD; status of vaccination against SARS-CoV-2; COVID-19-related characteristics (date of infection confirmed by polymerase chain reaction (PCR) or viral-antigen testing on nasopharyngeal swabs, symptomatology, imaging features, and COVID-19-related severity); information on oral antiviral agents (daily dose and administration time, and times of initiation and discontinuation); current immunosuppressive or immunomodulatory regimens during NR administration; sequential treatment regimens for patients who did not improve after using NR; outcome following NR administration and sequential treatments; time to symptom resolution; duration of hospital stay (DoHS); mortality; side effects; and interactions of NR with other drugs.

### 2.4 Definitions and grouping

We identified the “standard regimen” group as individuals who had mild-to-moderate symptoms and confirmed SARS-CoV-2 infection within 5 days and who received NR therapy in accordance with the latest guidelines and NR-prescribing information in China. These were also the typical conditions under which normative and standardization patients were administrated, thus deviation from this may be considered the non-standard regimen group. The “early NR regimen” group referred to people who received NR within 5 days of symptom onset, whereas the “late NR regimen”group received NR after 5 days. The National Medical Products Administration of China has approved NR use for the treatment of adult patients with mild-to-moderate COVID-19 and high-risk factors for progression to severe disease. If an adult patient with severe COVID-19 used NR, it was defined as an “NR non-indication regimen”. To account for potential confounding effects arising from a non-indication bias with NR, we conducted subgroup analyses that stratified patients with mild-to-moderate symptoms into an “early NR regimen” group and a“late NR regimen” group. To minimize the impact of the time of NR administration on patient outcome, we further categorized patients in the “late NR regimen” group into an “NR indication regimen” group and an “NR non-indication regimen” group.

Patient outcomes comprised the time of symptom resolution, DoHS, and response rate. The “time of symptom resolution” was defined as the time from symptom onset to improvement based on objective assessments: continuously decreasing temperature and no fever for >3 days; improved respiratory symptoms; obvious absorption of inflammation revealed on pulmonary imaging; negative PCR results on nasopharyngeal swabs. The “response rate” was defined as the proportion of symptoms improved at the first objective assessment after 5 days of NR use. “Comorbidities” referred to any pre-existing or concurrent medical conditions that occurred during the clinical course of RD.

### 2.5 Statistical analyses

Data are the median (interquartile range (IQR)) for continuous variables. Results are counts and percentages for categorical variables. Differences between groups using a standard regimen and non-standard regimen, early regimen and late regimen, as well as NR indication and NR non-indication regimens were analyzed by the chi-square test for categorical variables, and the Kruskal–Wallis test (as appropriate) for continuous variables. The cumulative probability of the response rate among patients at follow-up was calculated using Kaplan–Meier methods. “Follow-up” was defined as the interval from symptom onset to the first objective assessment of patient outcome after 5 days of NR use. The log-rank test was employed using Prism 9 (GraphPad, La Jolla, CA, United States of America) to assess disparities among groups for standard and non-standard regimens, early and late regimens, and NR indication and NR non-indication regimens, as well as groups of patients with ≥4 comorbidities and 4 comorbidities. Statistical analyses were undertaken using SPSS 25.0 (IBM, Armonk, NY, United States of America). *p* < 0.05 (two-sided) was considered significant.

## 3 Results

### 3.1 Characteristics of the participants

In total, 32 patients with RD infected with SARS-CoV-2 and who received NR between 8 December 2022 and 13 January 2023 were identified retrospectively. The characteristics of the population at baseline are shown in Table 1.

**TABLE 1:** Comparison of patients’ characteristics between the NR standard regimen and non-standard regimen groups.

Characteristics	Total (N = 32)	Standard regimen (n = 6)	Non-standard regimen (n = 26)	*P*
**Demographics**				
**Age years; n** (**%**)				
19-65	13 (40.6)	4 (66.7)	9 (34.6)	0.2
>65	19 (59.4)	2 (33.3)	17 (65.4)	
**Sex, n(%)**				
Male	10 (46.9)	1 (16.7)	9 (34.6)	0.6
Female	22 (53.1)	5 (83.3)	17 (65.4)	
**Anti-SARS-CoV-2 vaccine status**				
0 dose	29 (90.6)	5 (83.3)	24 (92.3)	0.5
≥1dose	3 (9.4)	1 (16.7)	2 (7.7)	
**Primary autoimmune disease diagnosis, n (%)**				
Systemic lupus erythematosus	11 (34.4)	1 (16.7)	10 (38.5)	0.6
Sjögren’s syndrome	9 (28.1)	2 (33.3)	7 (26.9)	1.0
Rheumatoid Arthritis	7 (21.9)	0	7 (26.9)	0.3
Dermatomyositis	4 (12.5)	1 (16.7)	3 (11.5)	1.0
Other disease	21 (65.6)	4 (66.7)	17 (65.4)	1.0
**Comorbidities, n (%)**				
Cardio-cerebrovascular disease	24 (75.0)	4 (66.7)	20 (76.9)	0.6
COPD and/or other chronic respiratory disease	21 (65.6)	2 (33.3)	19 (73.1)	0.1
Chronic renal disease	14 (43.8)	1 (16.7)	13 (50.0)	0.2
Diabetes mellitus	12 (37.5)	4 (66.7)	8 (30.8)	0.2
Chronic liver disease	12 (37.5)	0	12 (46.2)	0.1
Thyroid disease	11 (34.4)	2 (33.3)	9 (34.6)	1.0
Neurodevelopmental, neurodegenerative	6 (18.8)	3 (50.0)	3 (11.5)	0.1
Diseases				
<4 comorbidities	7 (21.8)	2 (33.3)	5 (19.2)	0.6
≥4 comorbidities	25 (78.1)	4 (66.7)	21 (80.8)	
**Previous Treatment, n(%)**				
**Glucocorticoids, n (%)**				
Prednisolone	21 (65.6)	3 (50.0)	18 (69.2)	0.4
**csDMARDS n, (%)**				
Hydroxychloroquine	11 (34.4)	2 (33.3)	9 (34.6)	1.0
Mycophenolate mofetil	8 (25.0)	2 (33.3)	6 (23.1)	0.6
Leflunomide	5 (15.6)	0	5 (19.2)	0.6
Cyclosporine	4 (12.5)	0	4 (15.4)	0.6
Other csDMARDS	10 (31.3)	0	10 (38.5)	0.1
**bDMARDS, n (%)**				
IL-2^3^	3 (9.4)	0	3 (11.5)	1.0
Rituximab	2 (6.3)	0	2 (7.7)	1.0
Other bDMARDS	3 (9.4)	0	3 (11.5)	1.0
**Botanical drug, n (%)**				
Triptergium wilfordii	2 (6.3)	0	2 (7.7)	1.0
Total glucosides of paeony	2 (6.3)	0	2 (7.7)	1.0
**Current treatment, n (%)**				
Dexamethasone	25 (78.1)	3 (50.0)	22 (84.6)	0.1
Human Immunoglobulin	19 (59.4)	3 (50.0)	16 (61.5)	0.7
Hydroxychloroquine	10 (31.2)	1 (16.7)	9 (34.6)	0.6
Prednisolone	5 (15.6)	1 (16.7)	4 (15.4)	1.0
Tocilizumab	5 (15.6)	1 (16.7)	4 (15.4)	1.0
Methylprednisolone	4 (12.5)	3 (50.0)	1 (3.8)	0.0*
**Sequential therapy, n (%)**				
Convalescent plasma infusion	5 (15.6)	0	5 (19.2)	0.6
Azvudine	3 (9.4)	0	3 (11.5)	1.0

NR: nirmatrelvir/ritonavir.

Other diseases included antiphospholipid syndrome, anti-neutrophilic cytoplasmic autoantibody (ANCA) vasculitis, gout, systemic sclerosis, immune thrombocytopenic purpura, autoimmune hemolytic anemia, polyarteritis nodosa, nodular nonsuppurative panniculitis, psoriasis1 and overlap syndrome.

Other csDMARDs, included cyclophosphamide, methotrexate, iguratimod, and tacrolimus.

Other bDMARDs, included belimumab, tumor necrosis factor inhibitor (TNFi), and tocilizumab.

*A two-sided *p*-value less than 0.05 was deemed to be a statistical difference.

The median age of all patients was 68 (IQR, 55–72) years and a higher proportion (59.4%) of patients aged >65 years were in the non-standard-regimen group. Most patients (n = 29/32, 90.6%) had not been vaccinated against SARS-CoV-2, and they were more commonly diagnosed with systemic lupus erythematosus (SLE, n = 11/32, 34.4%), Sjogren’s syndrome (SS, n = 9/32, 28.1%), and RA (n = 7/32, 21.9%).

The initial symptoms of COVID-19, in most cases, resembled those of other viral infections affecting the respiratory tract, typically dry cough (n = 20/32, 62.5%), fever (n = 18/32, 56.3%), and dyspnea (n = 16/32, 50.0%). The standard-regimen group had a lower prevalence of major comorbidities compared with that in the non-standard-regimen group, including cardio-cerebrovascular disease (66.7% vs. 76.9%), chronic obstructive pulmonary disease (COPD) and/or other chronic respiratory diseases (33.3% vs. 73.1%), chronic kidney disease (16.7% vs. 50.0%), and chronic liver disease (0% vs. 46.2%). Three deaths were observed (n = 3/26, 11.5%) in the non-standard-regimen group.

The immunosuppressive regimen administered previously primarily comprised glucocorticoids (n = 21/32, 65.6%), conventional synthetic disease-modifying anti-rheumatic drugs (csDMARDs, n = 22/32, 68.8%), biologic-DMARDs (n = 7/32, 21.9%), and other immunosuppressants (n = 4/32, 12.5%).

All patients diagnosed with COVID-19 received NR in addition to standard treatments according to the Clinical Guideline for COVID-19 Diagnosis and Treatment published by the National Health Commission of China (National Health Commission of the People’s Republic of China, 2023). There were three types of standard treatment. The first treatment type was dexamethasone (5–7.5 mg/day) or methylprednisolone (40 mg/day) for ≤ 7 days. The second therapy type was intravenous immunoglobulin (IVIG) and the dose was dependent on COVID-19 severity: mild = 100 mg/kg; moderate = 200 mg/kg; severe and critical illness = 400 mg/kg. The dose was administered ≤5 times. The third treatment type was tocilizumab. The initial dose of tocilizumab was 4–8 mg/kg, with a single dose ≤800 mg, and given not more than twice.

Subsequently, the most efficacious treatment regimens for patients given NR were dexamethasone (n = 25/32, 78.1%), IVIG (n = 19/32, 59.4%), and tocilizumab (n = 5/32, 15.6%). The frequency of methylprednisolone use was higher in the standard-regimen group compared with that in the non-standard-regimen group (*p* = 0.0).

In the non-standard-regimen group, eight patients who did not respond to NR therapy received convalescent plasma or azvudine therapy subsequently, and significant improvement was observed for all of them.

### 3.2 Patient outcomes between standard and non-standard regimens

**FIGURE 1:**
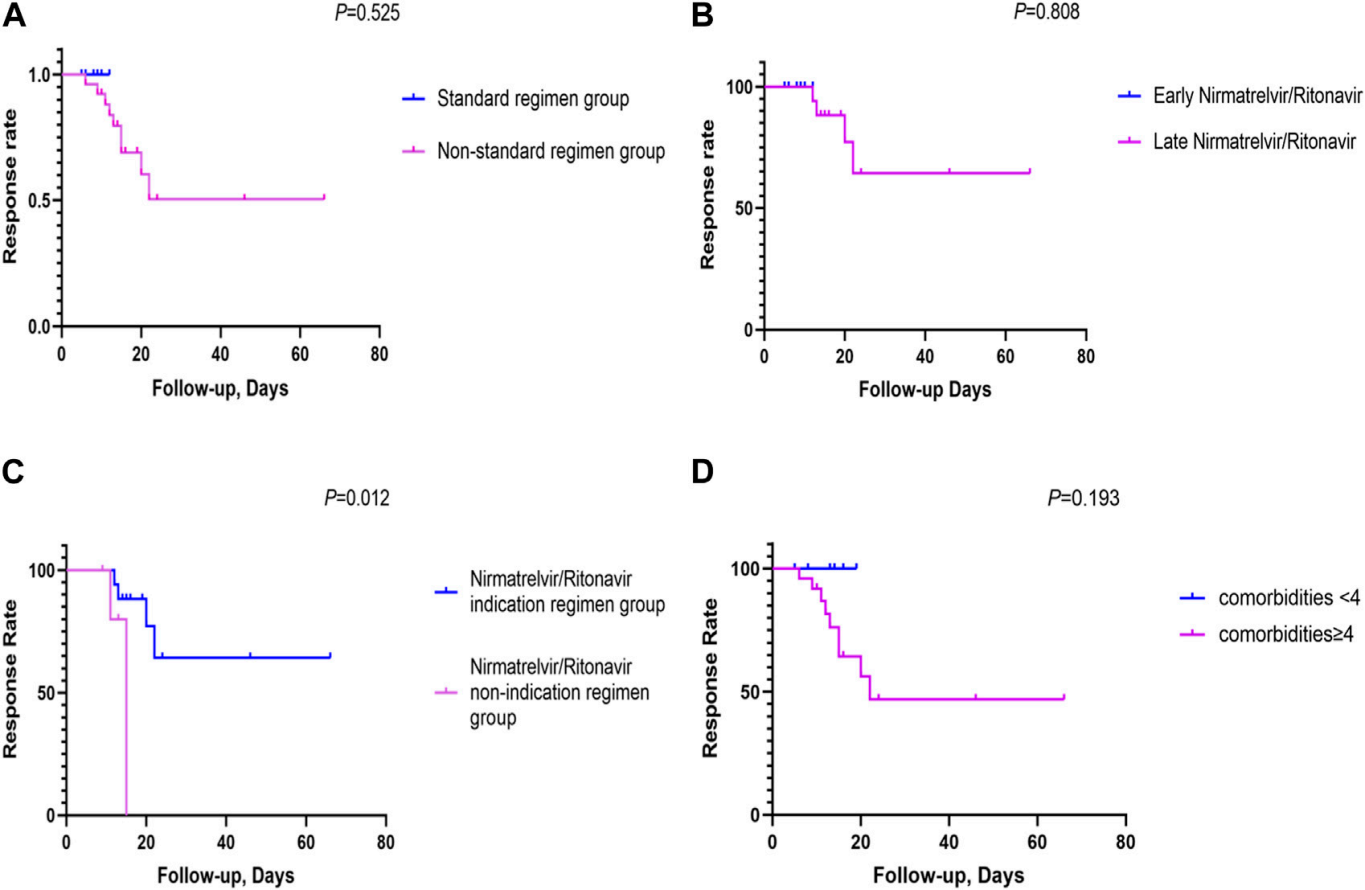
The cumulative probability of response rates between the different NR regimen groups **(A)** The cumulative probability of response rates between the NR standard regimen and the non-standard regimen groups (n = 32); **(B)** The cumulative probability of response rates between the early NR regimen group and late NR regimen group (n = 23); **(C)** The cumulative probability of response rates between the NR indication regimen group and the NR non-indication regimen group (n = 23); **(D)** The cumulative probability of response rates between comorbidities <4 and comorbidities≥4 (n = 32). (Kaplan-Meier survival curves were utilized to generate cumulative incidence curves for comparing patient response rates between different arms. The vertical axis denoted the overall response rate, and the horizontal axis represented the duration of follow-up. The follow-up period was defined as the interval from symptom onset to the first objective assessment of patient outcomes after 5 days of NR usage).

The standard-regimen group exhibited a shorter median time for symptom resolution in comparison with that in the non-standard-regimen group (9.0 (IQR 8.3–11.3) vs. 21.5 (IQR16.0–24.0) days) (*p* < 0.001). However, there was no significant difference in the median DoHS between the standard-regimen group and non-standard-regimen group (*p* = 1.0) (Table 2). Kaplan–Meier estimations revealed no significant differences (log-rank test, *p* = 0.5) in the cumulative probability of response between the standard-regimen group and non-standard-regimen group based on follow-up duration (Figure 1A). However, all symptoms improved in the standard-regimen group of patients at the end of follow-up.

**TABLE 2:** Comparison of patients’ outcomes between different NR regimen groups.

Patientoutcome	Total (n = 32)	Standardregimen (n = 6)	Non-standardregimen (n = 26)	*P*	Early NR (n = 6)	Late NR (n = 17)	*P*	NR indicationregimen YES,n (%) (n = 17)	NR non-indicationregimen NO,n (%) (n = 6)	*P*
Time to symptomresolution, days,median (Q1, Q3)	18.0 (13.8, 24.0)	9.00 (8.3, 11.3)	21.5 (16.0, 24.0)	0.0*	9.00 (8.3, 11.3)	23.0 (18.0, 24.0)	0.0^*^	23.0 (18.0, 24.0)	17.5 (14.5, 23.5)	0.4
Length of hospitalization, days, median (Q1, Q3)	14.0 (9.8, 19.5)	15.5 (10.3, 17.8)	13.5 (10.0, 20.5)	1.0	15.5 (10.3, 17.8)	12.0 (9.0, 15.0)	0.4	12.0 (9.0, 15.0)	20.0 (12.3, 33.8)	0.1

NR: nirmatrelvir/ritonavir; *A two-sided *p*-value less than 0.05 was deemed to be a statistical difference.

### 3.3 Patient outcomes between early and late NR regimens

We divided 23 patients with mild-to-moderate COVID-19 into groups of early NR regimen and late NR regimen. The early-NR-regimen group had a shorter median time of resolution compared with that of the late-NR-regimen group (9.0 (IQR 8.3–11.3) vs. 23.0 (IQR 18.0–24.0) days) (*p* = 0.0]. There was no significant difference in the median DoHS between the two groups (*p* = 0.4) (Table 2). NR administration within or beyond 5 days did not yield a significant impact on the response in those individuals (log rank test, *p* = 0.8). Nevertheless, a substantial proportion of patients (n = 13/17, 76.5%) continued to exhibit improved symptoms during the first objective assessment at the end of follow-up in the late-NR-regimen group (Figure 1B). All patients in the early-NR-regimen group showed symptom improvement at the end of follow-up.

### 3.4 Patient outcomes between NR-indication-regimen and NR-non-indication-regimen groups

We categorized the 23 patients receiving the late NR regimen into two subgroups based on whether they had indications for NR treatment or not. The time of symptom resolution was longer (23.0 (IQR 18.0–24.0) vs. 17.5 (IQR 14.5–23.5) days) (*p* = 0.4] in the NR-indication-regimen group compared with that in the NR-non-indication-regimen group. DoHS was shorter in the NR-indication-regimen group than that in the NR-non-indication-regimen group (12.0 (IQR 9.0–15.0) vs. 20.0 (IQR 12.3–33.8) days (*p* = 0.1) (Table 2). The proportion of patients who showed symptom improvement was higher in the NR-indication-regimen group compared with that in the NR-non-indication-regimen group (n = 13/17 vs. 3/6, 76.5% vs. 50.0%) at the end of follow-up. A significant difference (*p* = 0.0) was observed in the response of patients between the two groups (Figure 1C).

### 3.5 ADRs induced by DDIs

During the therapy course, all grades of ADRs and ADRs of grade ≥3 directly associated with NR administration were not observed in any of the 32 cases. Ten patients received hydroxychloroquine while one patient was administered sacubitril–valsartan and clopidogrel, both of which might interact with NR. However, interactions were not observed between NR and these three drugs. Despite discontinuing warfarin before NR application (NR was used on the first day of warfarin withdrawal), one patient experienced an increased international normalized ratio (INR; 5.32 (0.90–1.20)), prothrombin time (PT; 62.8 (9.4–12.5) s), and activated partial thromboplastin time (aPTT; 66.1 (25.1–36.5) s), as well as an increased fibrinogen level (425 (200–400) mg/dL) and coagulation disorders (weak positive fecal occult blood test) on the second day after using NR. The INR (1.25), PT (14.3 s), and aPTT (37.9 s) decreased to nearly normal values, and the fibrinogen level (345 mg/dL) and coagulation disorders returned to normal after 2 days of discontinuing NR (the seventh day after initial administration of NR).

### 3.6 Impact of comorbidities on patient outcome

Three deaths were observed in our study, one of whom had moderate COVID-19 and the other two had severe COVID-19. The treatment regimen for these three deceased individuals was NR, dexamethasone, and IVIG. The characteristics of the three deceased individuals were identified: age >65 years and additional comorbidities such as COPD and/or other chronic respiratory diseases (including interstitial lung disease (ILD) and pulmonary infection), cardio-cerebrovascular diseases (including arrhythmia, atrial fibrillation, coronary atherosclerosis, hypertension, and pulmonary hypertension), and type-2 diabetes mellitus. All of these ailments were risk factors for COVID-19 progression. We also analyzed the effect of comorbidities on the response to therapy. The percentage of patients who showed symptom improvement was higher in the group with <4 comorbidities compared with that in the group with ≥4 comorbidities (n = 7/7 vs. 16/25, 100.0% vs. 64.0%) at the end of follow-up. However, the number of comorbidities did not have a significant effect (*p* = 0.2) on the response rate between the two groups (comorbidities ≥4 or <4) (Figure 1D).

## 4 Discussion

We conducted this study in a real-world setting, which may differ from previous studies in terms of virus strains, study design, and settings. The EPIC-HR trial of NR was conducted during a period when the B.1.617.2 (Delta) variant was predominant in the United States (Hammond et al., 2022; Najjar-Debbiny et al., 2023). The present study was conducted at the beginning of the Omicron “wave” in China, during which the BA.5 and BF.7 variants were circulating predominantly and associated with a lower number of severe cases compared with those infected with the Delta variant. Importantly, we focused on vulnerable patient subgroups with RD undergoing therapy involving intensive immunodeficiency and/or immunosuppression. Patients with RD were typically receiving corticosteroids or other immunosuppressive drugs long-term. In our study, among 32 patients, 59.4% of individuals were aged >65 years, and 90.6% of patients had not received vaccination against COVID-19. All of these patients had received prednisolone and immunosuppressive drugs long-term, including hydroxychloroquine, mycophenolate mofetil, and biologic therapies (including rituximab) and 78.1% of patients had >4 complications (including cardio-cerebrovascular disease, COPD and/or other chronic respiratory diseases, and chronic kidney disease) (Gianfrancesco et al., 2020; Alzahrani et al., 2021; Arachchillage et al., 2022; Azizi et al., 2022; Sahebari et al., 2022). These are all risk factors for patients with RD suffering from COVID-19, which results in a potential hypofunctional immune state and puts them at high risk for severe COVID-19, hospitalization, and death. Furthermore, there may be a positive correlation between COVID-19 infection and prognosis with SLE, SSc, and RA. (Cordtz et al., 2021; Dewanjee et al., 2021; England et al., 2021; Grainger et al., 2021; Bournia et al., 2023). The expected response to SARS-CoV-2 vaccination for many patients with RD receiving systemic immunomodulatory therapies is blunted in its magnitude and duration compared with that in the general population but nonetheless emphasizes the importance of vaccination (Cordtz et al., 2022; Ammitzbøll et al., 2023; Curtis et al., 2023; Finckh et al., 2023). Moreover, other high-risk patients with RD who experience breakthrough infection (particularly among not fully vaccinated individuals infected with pre-Omicron variants) tend to have a worse prognosis (Bakasis et al., 2022; Liew et al., 2022).

The external environment, the characteristics of RD, and previous immunosuppressive therapies contributed to the particularity and complexity of our patients. Nevertheless, our data indicated that NR therapy was associated with a favorable outcome and an acceptable safety profile in a predominantly unvaccinated population with RD during the Omicron surge. The NR-standard-regimen group was associated with a lower risk of progression to severe outcomes in hospitalized patients with RD and COVID-19. Compared with the non-standard-regimen group, the standard-regimen group took less time for symptoms to resolve (Table 1) and more of them improved. All patients in the NR-standard-regimen group showed symptom improvement at the end of follow-up (Figure 1A). In this regard, the trend of our data was consistent with that in results reported in other real-world studies involving high-risk patients, RD population, and unvaccinated patients and the EPIC-HR trial (Wong et al., 2022a; Wong et al., 2022b; Hammond et al., 2022; Park et al., 2022; Aggarwal et al., 2023; Gentry et al., 2023; Kwok et al., 2023; Liu et al., 2023; Lui et al., 2023; Qian et al., 2023; Ramirez et al., 2023; Wan et al., 2023). Also, patients with RD infected with SARS-CoV-2 could benefit from early NR administration (within 5 days of symptom onset). The early-NR regimen reduced the time needed for symptom resolution to some extent, and all patients in the early-NR-standard-regimen group showed symptom improvement at the end of follow-up (Table 2; Figure 1A). The benefits to our patients align with a real-world study on early NR administration during the Omicron wave in high-risk patients (Bruno et al., 2022; Evans et al., 2023; Mutoh et al., 2023; Najjar-Debbiny et al., 2023; Yip et al., 2023).

Corticosteroids and some antirheumatic drugs may also be effective in treating COVID-19 (Raiteri et al., 2021; Rabie et al., 2022; Szekanecz et al., 2022). The number of cases taking other biologic drugs or csDMARDs was small and may have been insufficient to demonstrate other underlying effects (if present). We caution against causal inference regarding drug effects given the significant potential for residual confounding in our study. Fortunately, all patients diagnosed with COVID-19 in our study received NR in addition to standard treatments according to the Clinical Guideline for COVID-19 Diagnosis and Treatment (National Health Commission of the People’s Republic of China, 2023) (National Health Commission of the People’s Republic of China, 2023).

Furthermore, even if the duration of NR administration exceeded 5 days, patients with RD and mild-to-moderate COVID-19 who received NR could derive certain benefits from it (Figure 1C). NR has been approved by the US Food and Drug Administration for emergency use in adult patients with mild-to-moderate COVID-19 within 5 days of symptom onset and who are at a high risk of progression to severe disease. However, the efficacy of NR increased if it was administered in the first 24–48 h in the EPIC-HR trial (Hammond et al., 2022), which showed that treatment initiation within 5 days of symptom onset was associated with an 88% reduced risk of COVID-19-related hospitalization or death at 28 days. Our findings suggest that the NR-indication regimen enhanced improvement for patients with RD even if they experienced COVID-19 symptom onset beyond 5 days. A similar result was shown in a retrospective study from the multicenter EPICOVIDEHA registry in patients with a hematological malignancy, including those with symptom onset >5 days or patients with severe COVID-19 who continued to be administered NR (Salmanton-García et al., 2023), and an open-label, multicenter, randomized controlled trial including patients given NR within 5 days from symptom onset or a Ct value ≤ 25 of N and ORF1ab genes by real-time PCR (Liu et al., 2023). In conclusion, our results showed that early-NR or NR-indication treatment remained efficacious in patients with RD, and was a viable strategy for mitigating progression to severe disease. This awareness should prompt an immediate decision to use NR, thereby increasing the probability of disease improvement.

The percentage of patients who showed symptom improvement was higher in the group with <4 comorbidities compared with that in the group with ≥4 comorbidities. Patients in the non-standard-regimen group had more comorbidities, were older, and had a higher prevalence of cardio-cerebrovascular disease, COPD and/or other chronic respiratory diseases, as well as chronic renal disease, compared with the standard-regimen group. These are risk factors that indicate the progression of COVID-19 into a severe and critical stage (Gianfrancesco et al., 2020; Parohan et al., 2020; Azizi et al., 2022; Hammond et al., 2022; Mena-Vázquez et al., 2022; Najjar-Debbiny et al., 2023; Tiseo et al., 2023; Zhang et al., 2023), potentially resulting in a reduced efficacy of NR. Three deaths were observed in the non-standard-regimen group. All of these deceased patients were >65 years of age and had comorbidities such as ILD, COPD, pulmonary infection, arrhythmia, atrial fibrillation, coronary atherosclerosis, hypertension, pulmonary hypertension, and type-2 diabetes mellitus. Prolonged use of immunosuppressive agents in these three patients was also associated with an increased risk of death (Mena-Vázquez et al., 2022; Najjar-Debbiny et al., 2023). These findings emphasize the importance of physicians being aware of old age, immunosuppression, and comorbidities in patients with RD infected with SARS-CoV-2.

NR is a combination of nirmatrelvir and ritonavir. Nirmatrelvir is eliminated primarily by the kidneys, with minimal liver metabolism (Rizk et al., 2023). Utilization of ritonavir to enhance the plasma concentration of nirmatrelvir by inhibiting the expression of cytochrome P450 (CYP)3A4 confers a substantial potential for clinically significant DDIs (Lemaitre et al., 2023). The most important restriction of NR use is DDIs. Ritonavir is a potent inhibitor of CYP3A4, reaching maximal inhibition at a dose of 100 mg. Therefore, ritonavir can substantially increase the plasma concentrations of concurrently administered drugs metabolized predominantly by CYP3A4 (U S Food and Drug Administration, 2023; Marzolini et al., 2022; Rizk et al., 2023; Lemaitre et al., 2023). However, ritonavir exhibits moderate inhibition of CYP2D6 if administered as a “boosting dose”, and is an inducer of CYP1A2, CYP2B6, CYP2C9, and CYP2C19 enzymes (U S Food and Drug Administration, 2023; Marzolini et al., 2022; Rizk et al., 2023; Lemaitre et al., 2023). In addition, ritonavir inhibits the expression of the transporters P-glycoprotein and breast cancer resistance protein, which show high expression in the intestine, leading to the enhanced intestinal absorption of certain drugs. Ritonavir also hinders the hepatic uptake of organic anion transporting polypeptide (OATP)1B1 and OATP1B3, resulting in increased plasma concentrations of drugs such as statins (U S Food and Drug Administration, 2023; Marzolini et al., 2022).

Therefore, physicians and pharmacists should check the prescriptions given to patients. Also, the prescription should be adjusted while considering potential DDIs. For this reason (and also considering alleviation of immunosuppression), primary immunosuppressive or immunomodulatory treatments were discontinued before NR administration in our study. (Marsousi et al., 2018).

However, one patient received sacubitril–valsartan and clopidogrel, and 10 patients were administered hydroxychloroquine; these drugs could interact with NR. Sacubitril–valsartan and clopidogrel may have moderate interactions with NR (Dal-Ré et al., 2022; Eltayb et al., 2023). Sacubitril and valsartan have been reported to be substrates of human OATP1B1, OATP1B3, OAT1, and OAT3. Also, weak inhibition of the hepatic uptake transporter OATP1B1 by NR may increase the concentration of valsartan and the active metabolite of sacubitril, resulting in hypotension (U S Food and Drug Administration, 2023; Dal-Ré et al., 2022). Hence, blood pressure should be monitored upon co-administration with NR, and sacubitril–valsartan stopped if hypotension ensues (U S Food and Drug Administration, 2023; Abraham et al., 2022). Clopidogrel is a prodrug converted to its active metabolite by CYP3A4, CYP2B6, CYP2C19, and CYP1A2. Co-administration with ritonavir may reduce conversion to the active metabolite, leading to insufficient inhibition of platelet aggregation (U S Food and Drug Administration, 2023). One study assessed the combination of clopidogrel with ritonavir. The authors demonstrated that ritonavir reduced the area under the concentration–time curve by 3.2-fold (*p* = 0.02) and the maximum plasma concentration of a clopidogrel-active metabolite (*p* = 0.03), which led to diminished platelet inhibition (Ross et al., 2022). Thus, combined use of NR and clopidogrel should be avoided and, if possible, substitution with prasugrel should be attempted during NR treatment for ≥3 days (up to 5 days) after NR treatment (U S Food and Drug Administration, 2023). Significant DDIs are not expected between NR and hydroxychloroquine (U S Food and Drug Administration, 2023).

One patient was given NR on the first day after warfarin withdrawal and experienced increases in the INR, PT, aPTT, fibrinogen level, and coagulation disorders. Warfarin is a mixture of enantiomers. The S-enantiomer (more potent) is largely metabolized by CYP2C9. The R-enantiomer is metabolized by CYP3A4 and CYP1A2. Ritonavir inhibits CYP3A4 but induces CYP2C9 and CYP1A2 (U S Food and Drug Administration, 2023; Rizk et al., 2023). Reduction in warfarin exposure has been reported with chronic use of ritonavir, but an increase in warfarin exposure is anticipated with the short treatment course of NR because the onset of inhibition is more rapid than induction. The INR should be closely monitored if warfarin is administered with NR (U S Food and Drug Administration, 2023; Rizk et al., 2023; Ross et al., 2022). Despite discontinuing warfarin 1 day before NR application in one patient, an increase in the INR was observed, possibly due to the half-life of warfarin and the early, short treatment course of NR. Drug utilization for managing comorbidities may influence COVID-19 treatment with potential DDIs. This phenomenon highlights the safety concerns regarding DDI risks in COVID-19 management among patients with comorbidities.

Clinically evident adverse events related directly to NR were not observed. This observation may have been related to our relatively small patient cohort, which could have led to certain biases. In addition, patients with RD presented with more severe clinical manifestations of COVID-19, which may have obscured some mild ADRs.

## 5 Limitations

Our study had two main limitations. First, this was a retrospective, single-center cohort study focusing on a population with RD in a unique background during a special time period. Hence, the sample size might have been too small and/or the study period might have been too short to observe significant differences in some patient outcomes. Nevertheless, a discernible trend of clinical importance in patient outcomes was observed. According to the G-Power Calculator on http://www.gpower.hhu.de, the achieved power (1-beta error probability) from calculations was also compared with the desired power, which was set at 80% (Serdar et al., 2021). The calculated effect size of the NR standard regimen group was 1.0, while it was 4.6 in the non-standard regimen group. The common standard deviation (sigma, σ) for the NR standard regimen group was 2.6, and 12.1 for the NR non-standard regimen group. Using a two-sided *t*-test to detect the difference between two groups and setting the probability of type I error (alpha) at 0.05, the real sample size for the non-standard group was 26, and the power was 100.0% higher than the desired power. However, the real sample size of the standard regimen group was only 6 with a power of 57.0%, which is too small to be reliable. Nevertheless, other large-scale real-world clinical studies have also demonstrated the effectiveness of the standard NR regimen in preventing COVID-19 disease progression and our results may be still persuasive. Table 3 presents a comparison between the estimated sample size and the real sample size when the effect size is 0.2, 0.5, and 0.8 and the power value is 80% respectively. Second, with regard to the observational nature of our study, patients in the non-standard-regimen group were characterized by old age and a higher probability of comorbidities. These factors may have contributed to unfavorable outcomes and increased mortality rates. To overcome a potential selection bias and make the results more robust, we conducted subgroup analyses to evaluate the impact of the duration of NR administration, NR indication, and number of comorbidities on patient outcomes. Despite these limitations, our study provided interesting data on the use of NR > 5 days after symptom onset. Several factors limited the scope of our study. Fortunately, this study is only preliminary, and a large-scale clinical research study to verify the effectiveness of our method and veracity of our results would be worthwhile.

**TABLE 3:** Comparison of sample sizes between the estimated group and the real group.

Effect size	α	Power (%)	Estimated sample size group 1	Real sample size group 1	Estimated sample size group 2	Real sample size group 2
0.2	0.05	80	394	6	394	26
0.5	0.05	80	64	6	64	26
0.8	0.05	80	26	6	26	26

## 6 Conclusion

We showed NR therapy to be associated with a favorable outcome and an acceptable safety profile in an immunosuppressed population with RD during the Omicron surge. Early use of NR (within 5 days of symptom onset) could improve the prognosis of patients. NR administration for symptoms and confirmed SARS-CoV-2 infection after >5 days may also mitigate progression to severe disease and is a viable strategy. Our results highlight the importance of early utilization and/or NR indication, which may yield clinical advantages for patients with RD infected with SARS-CoV-2. Although our study shows interesting data on the use of NR more than 5 days after the onset of symptoms, it is worth conducting a large-scale clinical research study to verify the effectiveness of this method and its results.

## Data Availability

The raw data supporting the conclusion of this article will be made available by the authors, without undue reservation.
